# Network analysis reveals stage-specific changes in zebrafish embryo development using time course whole transcriptome profiling and prior biological knowledge

**DOI:** 10.1186/s13040-015-0057-1

**Published:** 2015-08-28

**Authors:** Yuji Zhang

**Affiliations:** 1 Division of Biostatistics and Bioinformatics, University of Maryland Greenebaum Cancer Center, Baltimore, USA; 2 Department of Epidemiology and Public Health, University of Maryland School of Medicine, Baltimore, USA

## Abstract

**Background:**

Molecular networks act as the backbone of molecular activities within cells, offering a unique opportunity to better understand the mechanism of diseases. While network data usually constitute only static network maps, integrating them with time course gene expression information can provide clues to the dynamic features of these networks and unravel the mechanistic driver genes characterizing cellular responses. Time course gene expression data allow us to broadly “watch” the dynamics of the system. However, one challenge in the analysis of such data is to establish and characterize the interplay among genes that are altered at different time points in the context of a biological process or functional category. Integrative analysis of these data sources will lead us a more complete understanding of how biological entities (e.g., genes and proteins) coordinately perform their biological functions in biological systems.

**Results:**

In this paper, we introduced a novel network-based approach to extract functional knowledge from time-dependent biological processes at a system level using time course mRNA sequencing data in zebrafish embryo development. The proposed method was applied to investigate 1α, 25(OH)_2_D_3_-altered mechanisms in zebrafish embryo development. We applied the proposed method to a public zebrafish time course mRNA-Seq dataset, containing two different treatments along four time points. We constructed networks between gene ontology biological process categories, which were enriched in differential expressed genes between consecutive time points and different conditions. The temporal propagation of 1α, 25-Dihydroxyvitamin D_3_-altered transcriptional changes started from a few genes that were altered initially at earlier stage, to large groups of biological coherent genes at later stages. The most notable biological processes included neuronal and retinal development and generalized stress response. In addition, we also investigated the relationship among biological processes enriched in co-expressed genes under different conditions. The enriched biological processes include translation elongation, nucleosome assembly, and retina development. These network dynamics provide new insights into the impact of 1α, 25-Dihydroxyvitamin D_3_ treatment in bone and cartilage development.

**Conclusion:**

We developed a network-based approach to analyzing the DEGs at different time points by integrating molecular interactions and gene ontology information. These results demonstrate that the proposed approach can provide insight on the molecular mechanisms taking place in vertebrate embryo development upon treatment with 1α, 25(OH)_2_D_3_. Our approach enables the monitoring of biological processes that can serve as a basis for generating new testable hypotheses. Such network-based integration approach can be easily extended to any temporal- or condition-dependent genomic data analyses.

**Electronic supplementary material:**

The online version of this article (doi:10.1186/s13040-015-0057-1) contains supplementary material, which is available to authorized users.

## Background

The active form of Vitamin D_3_ - 1α,25-dihydroxyvitamin D_3_ [1α,25(OH)_2_D_3_] – have demonstrated playing a critical role in calcium and phosphorus homeostasis by increasing intestinal calcium and phosphorus transport, thereby maintaining normal serum calcium and phosphorus concentrations and allowing bone mineralization to proceed [1, 2]. In previous works, we have shown that 1α,25(OH)_2_D_3_ alters expression of hundreds to thousands of genes at different developmental stages in early zebrafish embryos *in vivo* [3]. However, it is challenging to digest and interpret the regulatory relationships among these differentially expressed genes at adjacent developmental stages. Novel informatics approaches are needed to fill in the gap how to interpret these thousands of differentially expressed genes at different time points in a systematic manner.

Biological systems are highly dynamic and responsive to the external environment. The gene expression in these systems is a temporal process. Different genes are required to play different functional roles under different conditions. This is highly regulated by a complex regulatory system of diverse molecular interactions, such as protein-protein interactions (PPIs), protein-DNA interactions (PDIs), and metabolic signaling pathways [4]. Taking a snapshot of the gene expression profile in a biological system (e.g., cell cycle system and development) under a certain condition can reveal some of the genes that are specially expressed under this condition. However, to investigate how all the genes are regulated in the context of a biological system, and to determine the interaction relationships between these genes, it is necessary to measure the gene expression profile in a time series manner [5]. This can also provide the distinct possibility of unraveling the mechanistic drivers characterizing cellular responses [6]. Time series gene expression data have been widely applied to study a wide range of biological systems, including cell cycle [7], genetic interaction and knockouts [8, 9], and development [10]. Despite their unique features, many computational challenges still remain in analyzing such gene expression profiles. For instance, it is difficult to study the relationships among differentially expressed genes (DEGs) at each time point in a case–control time series experiment, due to large number of DEGs and limited time points available. To address such challenges, algorithms are required that are specifically designed to improve the interpretability of these data by integrating multi-source prior biological evidence.

Molecular interactions such as PPIs and PDIs are essential for a wide range of cellular processes and form a network of astonishing complexity. Until recently, our knowledge of such complex networks was rather limited. The emergence of high-throughput technologies has given us possibilities to systematically survey and study the underlying biological system. The molecular interaction maps have been built in model organisms (e.g., *S.cerevisiae* [11], *D.melanogaster* [12] and *C.elegans* [13]), as well as in higher vertebrate organisms (e.g., zebrafish [14], mouse [15] and human [16]). Evidently, the generated interaction maps offer us a rich resource for systematic studies of molecular networks and complement other types of biological data. However, current interaction databases include a large amount of false positive and false negative interactions due to the unreliability of interaction mapping technologies available. In addition, these molecular interactions are static. There is little direct information available on the temporal dynamics of these molecular interactions. To understand time-dependent biological processes at network level, molecular networks need to be considered as temporal and spatial rather than static information flow between molecules [17]. Recently, attempts have been made in integrating different types of biological data with molecular network interactions to reveal the dynamics of molecular networks [18]. However, only a few studies have investigated the dynamics of the molecular network interactions in time course gene expression data with limited success. For instance, Tang et al. [19] proposed to reconstruct time course protein interaction networks (TC-PINs) by incorporating time series gene expression into PPI networks. The functional modules from TC-PINs were enriched in related gene ontology (GO) biological processes than those from static PPI networks. However, the causal relationship between TC-PINs across time points could not be inferred. Such causal relationships are crucial to understand the underlying regulated biological processes in a time-dependent and context-specific manner. A propagation of such interactions from gene level to biological process/pathway level (e.g., gene ontology information) will help us identify the altered biological processes during the time in which these gene expressions are examined.

The gene ontology (GO) Consortium [20] has developed three separate ontologies-molecular function (MF), biological process (BP) and cellular component (CC) - to describe the attributes of gene products. Several studies have demonstrated that the molecular interactions and GO provide substantially congruent yet subtle different view of biological systems [21]. The hypothesis is that the interaction between any two proteins/genes indicates a general likelihood that these two proteins are functionally coupled or involved in the same biological process. Identifying enriched interactions between any two GO terms based on molecular interactions between genes assigned to these two GO terms are more statistically reliable: interactions reflect statistically enriched temporal connections between multiple genes of one GO term and multiple genes of another. However, this could not tell the temporal directionality in these connections. By incorporating time series gene expression data, the causal relations can be inferred in this GO network by highlighting information flow between GO biological processes enriched in DEGs at consecutive time points.

**Fig. 1:**
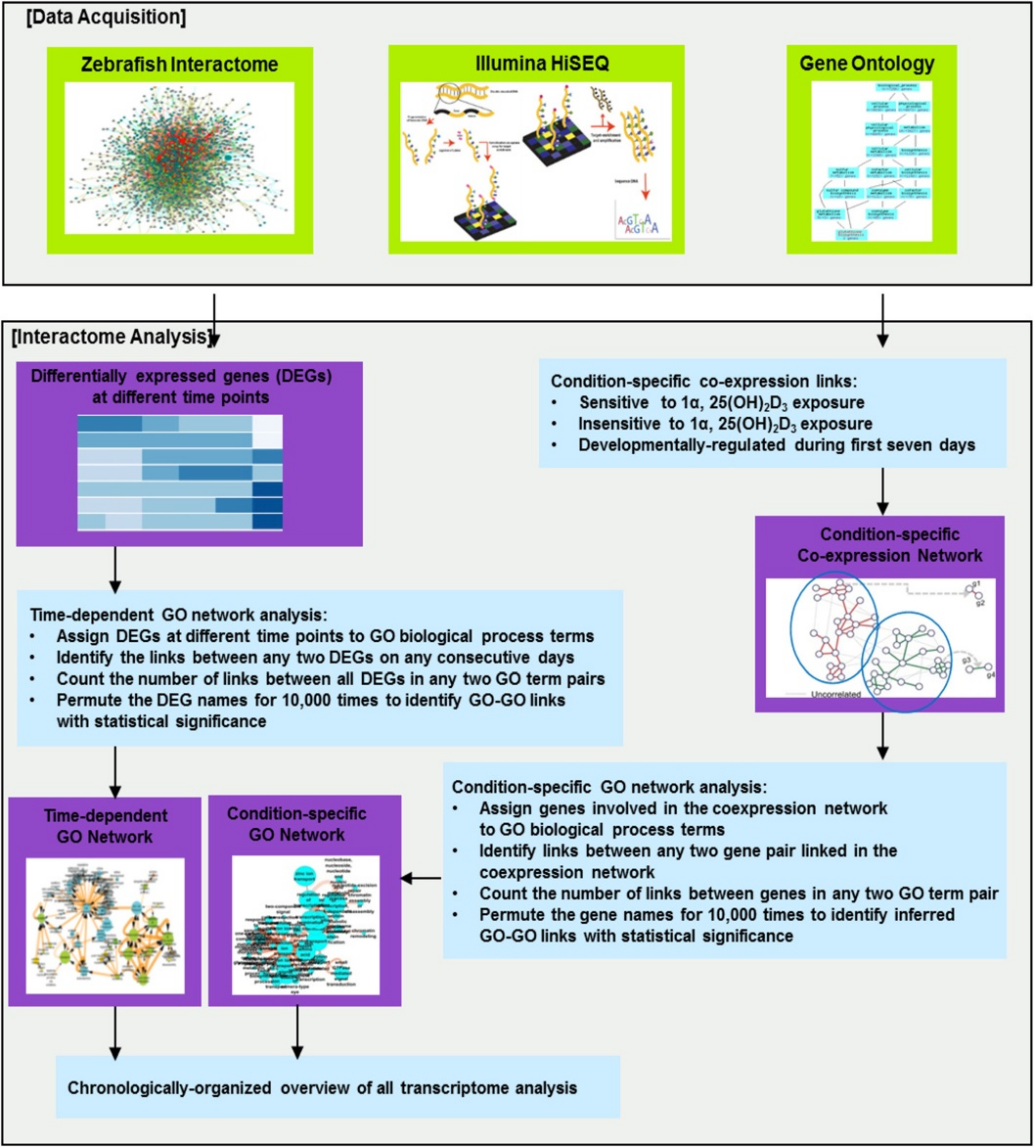
Overview of the proposed approach

In this paper, we developed a novel network-based computational approach to study causal relationships between DEGs at consecutive time points in a case–control time series experiment. To overcome the limitation that the intervals of time series experiments usually would not fit the time scale of functional communications between most genes and the statistical power from only several time points would be too low for robust analysis, we constructed networks of GO biological process terms connected by significant interactions between DEGs on sequential time points. This enables us to understand the biological processes at GO scale, in which relations between nodes (representing GO terms) are more statistically stable. This is more statistically significant and biologically meaningful compared to single co-expressed links. The detail of the proposed approach is presented in Fig. 1. The proposed method was applied to time series mRNA-Seq data set to determine the influence of 1α,25(OH)_2_D_3_ treatment on gene expression patterns in zebrafish embryo development and the causal relationship between DEGs at consecutive time points. The resulting networks suggest that well-studied as well as novel molecular mechanisms are regulated by 1α,25(OH)_2_D_3_ treatment.

## Results

In this section, we present: (1) a description of generation and initial characterization of the mRNA-seq dataset obtained from zebrafish embryos altered by 1α,25(OH)_2_D_3_ treatment; (2) an overview the interactome-based analysis that we proposed; (3) a chronologically organized analysis of the transcriptome changes and interactome dynamics altered by 1α,25(OH)_2_D_3_ treatment during early zebrafish development. Figure 1 illustrates the overview of the proposed analysis workflow.

### Characterization of mRNA-seq dataset during zebrafish embryo development

Genome-wide transcriptional profiling were performed using Illumina HiSeq sequencing technique for four replicate cDNA libraries of 1α,25(OH)_2_D_3_- or vehicle-treated zebrafish, 48, 96, 144, and 168 hours post fertilization (hpf) as described in our previous publication [3]. Overall, the RNA-seq data obtained from 32 independent zebrafish RNA libraries had comparable number of total reads [3]. These reads were mapped to the latest zebrafish genome assembly (zv9) from the UCSC website (http://genome.ucsc.edu/). The refFlat annotation file from the University of California Santa Clara (UCSC) Table Browser was used to generate raw reads mapped to each annotated gene in the annotation file. The genes altered by 1α,25(OH)_2_D_3_ treatment at each time point were identified using the negative binomial model as describe in [22]. A list of altered genes identified along with the days on which they were differentially expressed is presented in Additional file 1: Table S1. We also carried out the gene ontology (GO) enrichment analysis using the GOMiner tool [23]. However, due to the limited number of DEGs identified at each time point and the limitation associated with the Fisher’s Exact Test, the results of these analyses could not provide much indication of the biological processes being modulated in response to 1α, 25(OH)_2_D_3_ treatment. To more efficiently derive biological insights from the genome-wide transcriptomic response to the treatment, we proposed a network-based analysis in the following sections.

### Interactome-based analysis of differentially expressed genes during zebrafish development

We overlaid the DEGs onto the zebrafish functional interactome from the FunCoup database [14]. The DEGs were overlaid on their corresponding nodes in the interactome, and related functional interactions between genes were extracted and reconstructed the 1α,25(OH)_2_D_3_ specific interactome. Many network interactions connect the few genes altered on day 2 and many altered on later days. We found that there was a statistically significant enrichment in links between genes that were 1α, 25(OH)_2_D_3_-altered earlier and genes regulated later in the course of experiment. This suggested that treatment affected signals were propagated along network routes from the initially affected genes (on day 2) towards network regions that were perturbed later.

**Fig. 2:**
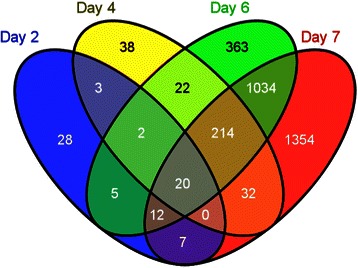
Venn diagram showing the overlap of DEGs at different developmental stages. Genes were grouped based on the day(s) they were differentially expressed. In four studied developmental stages 3134 genes were defined as differentially expressed during at least one stage

Specifically, 3134 genes were up- or down-regulated by 1α,25(OH)_2_D_3_ on at least one of the four days in the experiment (adjusted P value less than 0.01). On day 2, only 77 genes were changed. 331 genes on day 4, 1672 genes on day 6, and 2673 genes on day 7 differentially expressed in response to 1α,25(OH)_2_D_3_ treatment (Fig. 2). The property of these DEGs was investigated in the context of FunCoup network. The average degree of DEGs is significantly higher than non-DEGs (14.9 versus 5.8, the P value of one-way ANOVA less than 10^−6^). This indicated that DEGs were more enriched in hub genes (genes with higher node degree). This can partially explain the initially altered genes on days can pass the changes to more interacted genes on later days through the network links/interactions.

To gain a better perspective on what this temporal pattern in enriched connections between 1α,25(OH)_2_D_3_-altered genes might mean, we analyzed the GO categories associated with the connected nodes in the context of interactome.

### Network propagation analysis of differentially expressed genes during zebrafish development

The FunCoup network links among these genes can indicate a general likelihood how they are functionally related, but don’t highlight the temporal directionality in these connections. Causal relations can be suggested by examining temporal changes, i.e., if information associated with gene *A* at time point *t* helps to predict the state of gene *B* at time point (*t* + 1), then a causal relation A- > B might be inferred [24, 25]. However, traditional network inference approaches could not identify such temporal regulatory relationship due to limited time points available. The statistical power from only four time points would be too low for robust analysis. To gain a better perspective on the temporal pattern among 1α, 25(OH)_2_D_3_-altered genes, we generalized a network of GO terms connected by the links between these DEGs on consecutive time points. At this broader scale, relations between nodes (GO biological processes) are statistical reliable: links reflect statistically enriched temporal connections between multiple genes of one node with multiple genes of another. Thus, this GO-GO network highlights flow between GO biological processes altered by 1α, 25(OH)_2_D_3_ on different days.

**Fig. 3:**
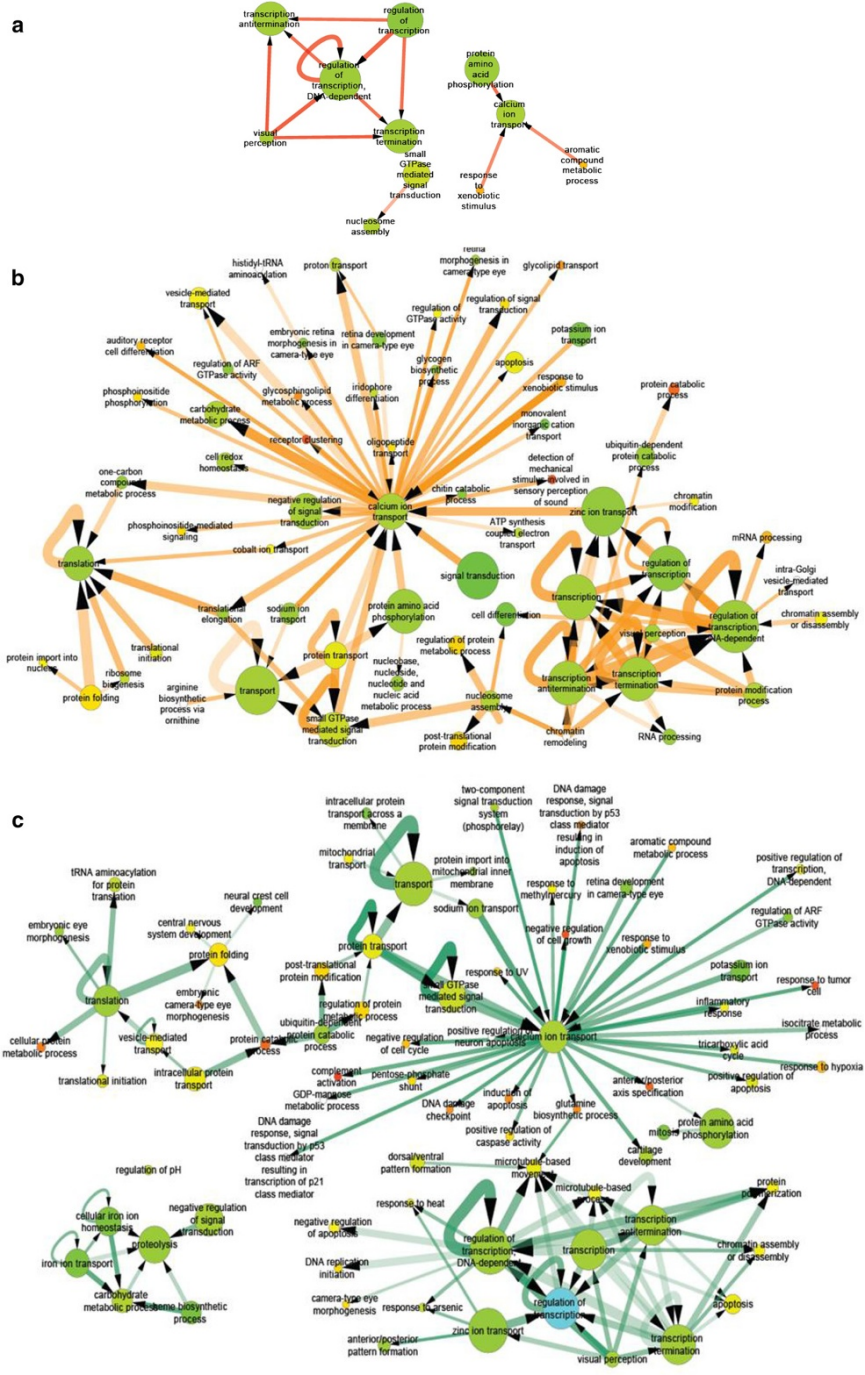
Network of GO terms enriched in 1α,25(OH)_2_D_3_ -altered genes between consecutive days. **a** GO network of day 2 - > 4; **b** GO network of day 4 - > 6; **c** GO network of day 6 - > 7. Color represent the fraction of the gene in that node that were regulated by 1α,25(OH)_2_D_3_ on any day (green is low and red is high). Edge thickness and opacity represent the number of gene-gene links between two GO terms and significance score (−log_10_(*P* value)), respectively

**Fig. 4:**
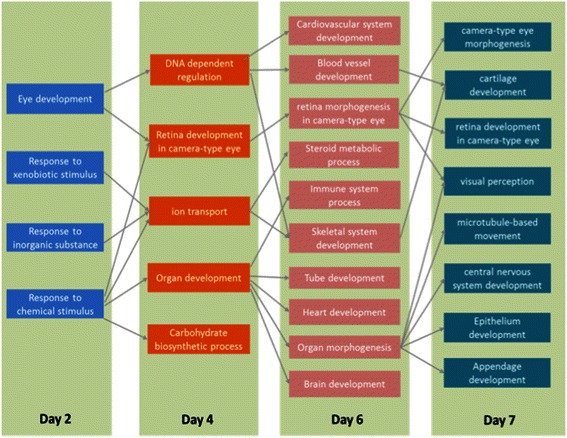
Overview of associations among GO terms enriched in 1α,25(OH)_2_D_3_ -altered genes at each time point

1α, 25(OH)_2_D_3_-altered genes in individual gene-gene interactions in FunCoup interactome were labeled with days when these genes were detected as differentially expressed. We were particularly interested in identifying the links in which one gene was altered earlier than the other. Thus, if there were a significant number of genes in GO category *X* altered on day *d* interacting with gene in GO category *Y* altered on day (*d* + 1), we hypothesize that a causative relation *X* - > *Y*. Limiting the output to only enriched GO-GO connections allowed us to focus on the major changes of propagation of 1α, 25(OH)_2_D_3_ and organismal response to it. Compared to the individual category enrichment approach such as GOMiner, our approach yielded a much richer analysis for interpretation of time series changes unique to time series gene expression data. The Figs. 2–4 presented day-to-day enriched interactions at GO biological process level. We provided a chronological interpretation on these findings below.

### Chronological analysis of the interaction network altered by 1α, 25(OH)_2_D_3_ at gene ontology level

The approach described above enabled flexible and deep monitoring of 1α, 25(OH)_2_D_3_ altered changes in the transcriptome at GO level in the context of functional interactome. To show time-dependent information flow in embryonic development altered by 1α,25(OH)_2_D_3_ treatment, GO networks of enriched GO-GO interactions were reconstructed.

#### Day 2 to day 4 transition

The network of GO terms between DEGs on day 2 and 4 suggested a cascade initiated by changes in xenobiotic metabolism genes and leading to genes involved in ion transport and transcription regulation (Fig. 3(a)). The “eye development” category is enriched on as early as day 2, indicating that eye development was changed by 1α, 25(OH)_2_D_3_ treatment. The eye development of zebrafish starts as early as 28 hpf [26]. The vitamin D receptor has been shown to express in various tissues and organs including retina. This confirms the finding using our proposed approach.

#### Day 4 to day 6 transition

Day 4 was marked by the most significant increase of linkage from transcription factors altered on day 4 towards others altered later. The most central node on day 4 is organ development, which became connected to multiple biological processes, such as cardiovascular system development, blood vessel development, immune system process, heart development, brain development, tube development, and others. This observation suggests that vitamin D treatment can alter biological processes involved in the development of many organs. One network of GO terms between day 4 and 6 is presented in Fig. 3(b).

#### Day 6 to day 7 transition

The organ morphogenesis was identified as a central node in the GO network of day 6 - > 7, connecting to multiple biological processes, such as nervous system development, circulatory system development, vasculature development, epithelium development, retina development in camera type eye, and many embryonic development terms including cartilage development and neuron generation. One network of GO terms between day 6 and 7 is presented in Fig. 3(c).

To better interpretate the causal relationships between enriched GO categories on consecutive days, we presented a GO level information flow by combining the GO-GO networks across all four days (Fig. 4). The interactome was altered in the regions scattered in the interactome to many biological processes that are clustered together in the interactome. This suggests that the effect of 1α, 25(OH)_2_D_3_ treatment can be as early as 48 hpf in early zebrafish development.

### Condition-specific GO network analysis

**Fig. 5:**
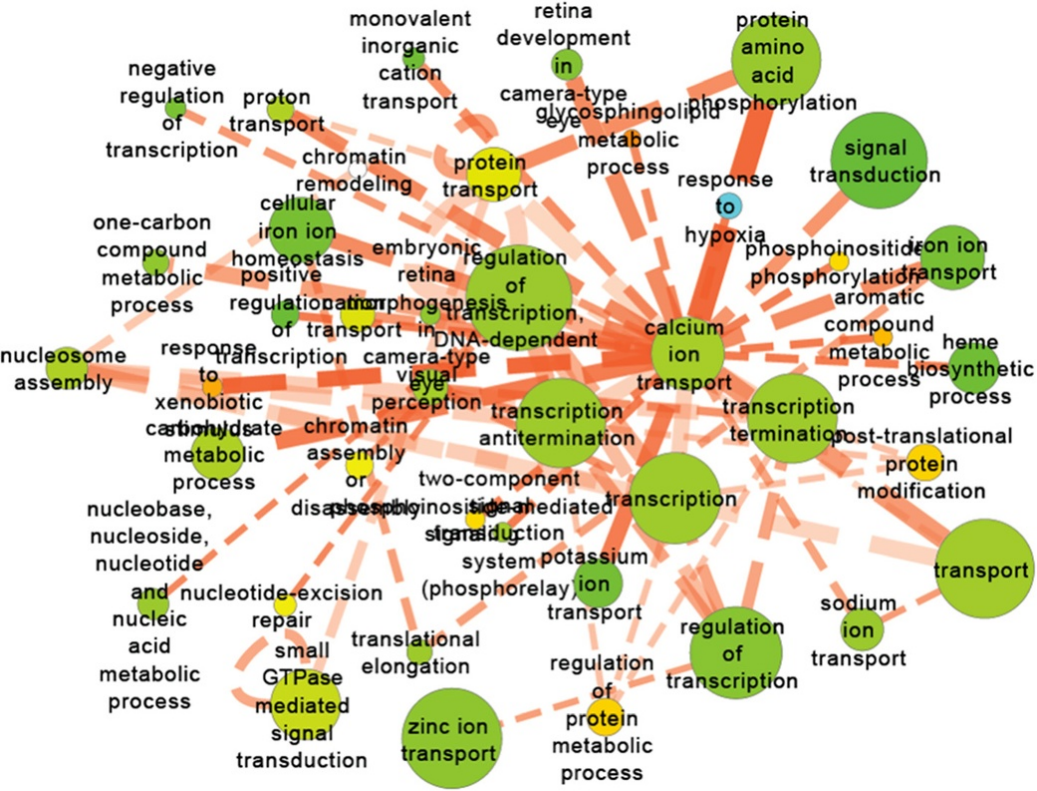
Network of GO terms enriched in 1α,25(OH)_2_D_3_-specific co-expression links. Node color represent the fraction of the gene in that node that were regulated by 1α,25(OH)_2_D_3_ on any day (green is low and red is high). Edge thickness and opacity represent the number of gene-gene links between two GO terms and significance score (−log_10_(P value)), respectively. Dotted line represents enriched co-expression relationships between genes

We also constructed the condition-specific meta-flow network based on co-expressed links identified. The statistics of three types of condition-specific links is presented in Table 1. A meta-flow network of GO terms was constructed for each condition-specific coexpression network. For instance, in 1α,25(OH)_2_D_3_-specific GO network (Fig. 5), several calcium metabolism-related GO terms were inferred by our approach including calcium ion transport, one-carbon compound metabolic process. Specifically, the response to hypoxia has been reported to be inhibited by 1α,25(OH)_2_D_3_ in human cancer cells [27]. In addition, there were quite a few developmental-related processes altered by 1α,25(OH)_2_D_3_ treatment, such as translation elongation, nucleosome assembly, and retina development. All these enriched GO terms indicated that 1α,25(OH)_2_D_3_ altered several pathways in developing eukaryotes.

**Table 1:** Statistics of co-expressed links

Link type	Gene #	Link #	Co-expressed link
1α, 25(OH)2D3–related	4025	13945	5622
Ethanol–related	4233	14590	5321
Developmental	2245	10344	2432

## Discussion

In this work, we have developed a network-based computational approach that analyzes time series mRNA-seq gene expression profiles in the context of molecular interactome and GO information to reveal temporal transcriptional changes altered by 1α,25(OH)_2_D_3_ in zebrafish embryo development. This enabled us to review the progression of 1α,25(OH)_2_D_3_-induced changes in gene expression and the network structure itself in zebrafish embryo development. The efficiency of our analysis of 1α,25(OH)_2_D_3_-alered global gene expression was enhanced by the interactome approach, as the network-based analysis approach were superior to their single-gene approach in terms of both statistical power and biological interpretability, A variety of interesting biological hypotheses were derived from our analysis. The significant biological processes include iron metabolism, neuronal and retinal development, and many organ development related pathways. Our approach is useful for discovering candidate biological processes that can serve as a basis for generating new testable hypotheses. Such network-based integration approach can be extended to any temporal- or condition-dependent genomic data analyses. Other types of interaction or ontology data can also be incorporated into this approach.

## Conclusions

We have developed a network-based analysis approach that integrated mRNA-seq gene expression profiles with molecular network and GO annotation to reveal dynamic propagation of 1α,25(OH)_2_D_3_-altered transcriptional changes from a few genes that altered initially, to large groups of biologically coherent genes at later times. The most notable biological processes included calcium and iron metabolism, neuronal and retinal development, and generalized stress response. Such network-based integration approach can be extended to other condition-dependent studies. Also graph theory can be incorporated to compare condition-specific coexpression networks and meta-flow networks of GO terms can be inferred based on such information.

## Material and methods

### mRNA-Seq gene expression data

The mRNA-Seq profiling in four biological replicate samples of 1α,25(OH)_2_D_3_- or ethanol-treated zebrafish, 2, 4, 6 and 7 days post-fertilization (hpf) was obtained by the Illumina HiSEQ 2000 platform. The generated 50-bp FASTQ sequence reads were aligned to both the latest Zebrafish genome assembly (zv9) and our in-house exon junction database using BWA [28]. The aligned sequence tags were counted for each annotated genes/exons using custom scripts based on the UCSC genome binning approach [29]. A total of 14267 genes were annotated using RefSeq database and the raw read counts for genes were generated for further downstream analyses.

### Zebrafish molecular interaction network

The zebrafish molecular interaction network was downloaded from FunCoup database (http://FunCoup.sbc.su.se/). In total, there are 1,999,529 interactions between 13033 proteins in the zebrafish interactome downloaded on January 3rd, 2012.

### Gene ontology annotation in zebrafish

The gene ontology annotation was downloaded from the original website (http://www.geneontolgy.org/) on Januray 20th, 2012. In this paper, we used the biological process terms only since our goal is to identify the 1α,25(OH)_2_D_3_-altered mechanisms.

### Differential gene expression analysis

For differential gene expression analysis between conditions, we eliminated genes without any reads across all samples. We used DESeq package in R to test for differential expression for all the remaining genes [22]. We conservatively accounted for multiple testing, employing a Bonferroni correction, and yielding an adjusted p-value for differential expression for each gene. A strict adjusted P value cut-off of 0.01 was used to select significant DEGs.

### Construction of time-dependent GO-GO networks

A network of GO terms was generalized from the network of DEGs at different developmental stages in zebrafish embryos. At GO scale, relations between nodes (representing GO terms) are more statistically stable. Links reflect statistically enriched temporal connections between multiple genes in one specific GO term and multiple genes in another one. Thus, this GO-GO network highlights information flow between GO biological processes affected by 1α,25(OH)_2_D_3_ at different developmental stages. If there were a significant number of genes in GO term X first altered at one time point interacting with genes in GO term Y first altered on the next time point, we hypothesize that a causative relation exists X - > Y. We limited the network to only enriched GO-GO connections, i.e. one with significant more gene-gene interactions (given both genes were 1α,25(OH)_2_D_3_-altered) than expected by chance. This allows us to focus on the major tendencies of propagation of 1α,25(OH)_2_D_3_ treatment and organismal response to it. Compared to the individual category enrichment, this approach yielded a much richer analysis for interpretation. The detailed reconstruction step is as follows:

1. For any two GO terms, a link was counted if any two DEGs in these two GO terms were connected in the original FunCoup network;
2. The GO-GO links were classified into time-dependent patterns according to the days when the gene were differentially expressed for the first time:
  - Day 2 - > Day 4: one gene was differentially expressed on Day 2, while the other on Day 4;
  - Day 4 - > Day 6: similar definition as in (a);
  - Day 6 - > Day 7: similar definition as in (a).
3. The GO For each candidate GO-GO network link, its statistical significance was evaluated by the permutation test, i.e. gene names were randomized in the FunCoup network for 10,000 times. The links between GO terms with P value less than 0.01 were considered statistically significant.
4. Enriched GO-GO links were kept in the GO-GO network, i.e. ones with P value less than 0.01. The network was visualized in the Cytoscape tool [30].

### Construction of condition-specific co-expressed interaction networks

To obtain the condition-specific expression information, a network called the co-expressed interaction network (CEIN) was constructed. Correlation of gene expression profiles between each pair of interacting proteins in FunCoup was evaluated by Pearson correlation coefficient (PCC). PCC of paired genes *X* and *Y*, which encodes one pair of interacting proteins, is defined as

1 $$ PCC\left(X,Y\right)=\frac{1}{n-1}{\displaystyle \sum_{i=1}^n\left(\frac{X_i-\overline{X}}{\sigma (X)}\right)\left(\frac{Y_i-\overline{Y}}{\sigma (Y)}\right)} $$

where *n* is the number of condition-specific samples; *X*_*i*_ and *Y*_*i*_ is the expression level of gene *X*(*Y*) in the sample *i* under a specific condition (1α, 25(OH)_2_D_3_ or ethanol treated); $\overline{X}$ ($\overline{Y}$) represents average expression level of gene *X* (*Y*) and *σ*(*X*) *σ*(*Y*)) represents the standard deviation of expression level of gene *X* (*Y*). Large absolute value of PCC indicates higher correlation between two gene pair evaluated. Besides correlation relationship, when applied to a pair of gene expression profiles, the experimental design allowed measuring effects of factors “1α, 25(OH)_2_D_3_ treatment”, “developmental stage”, and “gene” as well as any of their combinations. The procedure was executed under the terms of the standard 3-way factorial ANOVA. By combining PCC and ANOVA analyses, we defined three types of coexpression networks:

- 1α, 25(OH)_2_D_3_ – related coexpression network with strong correlation between observed gene expression profiles only after 1α, 25(OH)_2_D_3_ treatment;
- Ethanol – related coexpression network with strong correlation between observed gene expression profiles only in ethanol treatment;
- Developmental - related coexpression network with strong correlation between observed gene expression profiles under both conditions and with a significant developmental pattern and synchronous between two genes.

The first two types of coexpression links were assigned if the following conditions hold:

2 $$ \max \left(\left|PC{C}_{VD3}\left|,\right|PC{C}_{ethanol}\right|\right)>\mathrm{m}\mathrm{i}{\mathrm{n}}_{PCC} $$

3 $$ \frac{\left|PC{C}_{VD3}-PC{C}_{ethanol}\right|}{ \max \left(\left|PC{C}_{VD3}\left|,\right|PC{C}_{ethanol}\right|\right)}>dif{f}_{PCC} $$

4 $$ \min \left({F}_{TREAT},{F}_{TREAT* GENE},{F}_{TREAT* GENE* DAY}\right)>{F}_{\alpha =0.05;1,19} $$

where *PCC*_*VD3*_ refers to the PCC value for the 1 α, 25(OH)_2_D_3_-treated samples, and *PCC*_*ethanol*_ refers to the PCC value for the ethanol-treated samples. Eq. (2) insures that at the least one of the PCC values exceed the threshold min_*PCC*_, while Eq. (3) requires that the difference between two PCC values in different conditions is big enough, i.e., larger than *diff*_*PCC*_. Eq. (4) states that at least one of the three effects from ANOVA analysis must be significant (i.e. *P* <0.05).

The third type of coexpression link was assigned given all the following conditions hold:

5 $$ PC{C}_{all}>{full}_{PCC} $$

6 $$ {F}_{DAY}>{F}_{\alpha =0.05;3,19} $$

7 $$ {F}_{DAY* GENE}<{F}_{\alpha =0.2;3,19} $$

where *PCC*_*all*_ refers to the PCC value for all samples across all conditions, and *full*_*PCC*_ is the minimum PCC value for a link to be considered coexpressed. In this paper, we set the cutoff values 0.9, 0.6, 0.9 for min_*PCC*_, *diff*_*PCC*_ and *full*_*PCC*_.

### Construction of condition-specific GO-GO networks

To generate the condition-specific GO-GO network view, a condition-specific network of GO categories was reconstructed. It was based on the genes that were involved in condition-specific network (e.g. 1α, 25(OH)_2_D_3_-sensitive coexpression network) and assigned to at least one GO biological process. The reconstruction step is as follows:

1. For any two GO “biological process” categories, a link was counted if any two genes in these two GO categories were connected in the condition-specific coexpression network;
2. For each potential GO-GO network link, its statistical significance was evaluated by the permutation test, i.e. gene names were randomized in the co-expression network for 10,000 times. The links between GO biological process terms with P value less than 0.01 were considered statistically significant.

Enriched GO-GO links were kept in the GO-GO network, i.e. ones with *P* value less than 0.01. The network was visualized in the Cytoscape tool.

### GoMiner analysis

The gene level Gene ontology enrichment analysis was performed using GoMiner [23] on the DEGs that were identified at each time point.

## Additional file

Additional file 1: **A list of altered genes identified along with the days on which they were differentially expressed.** (XLS 1332 kb)
